# Urinary neutrophil gelatinase-associated lipocalin as an early predictor of acute kidney injury in cardiac surgery patients

**DOI:** 10.1186/cc12349

**Published:** 2013-03-19

**Authors:** T García Rodríguez San Miguel

**Affiliations:** 1 Hospital Santa Creu i Sant Pau, Barcelona, Spain

## Introduction

To evaluate whether urinary neutrophil gelatinase-associated lipocalin (uNGAL) detects acute kidney injury (AKI) earlier than the estimated glomerular filtration rate (eGFR) in cardiac surgery patients.

## Methods

Two-hundred and seventy-four adult patients undergoing cardiac surgery were consecutively included from February to December 2011. Exclusion criteria were absence of diuresis due to end-stage renal disease or chronic renal failure and a previous cardiac catheterism with i.v. contrast use the week before surgery. Four serial blood and urine samples immediately before (PRE) and after (POST) surgery, and 1 days (1d) and 2 days (2d) after surgery were obtained. uNGAL was measured in an Architect 6200 (Abbott Diagnostics). AKIN criteria were used to diagnose AKI. The study was approved by the local ethics committee and all patients gave informed consent. Delta uNGAL was defined as the difference between the PRE and the POSTs concentrations.

## Results

One-hundred and eighty-one patients (66.1%) were men; mean age was 68.2 ± 12.2 years. Valve replacement was performed in 123, coronary artery bypass graft (CABG) in 81, valve surgery + CABG in 48, cardiac transplant in five, aorta aneurism surgery in nine, and other procedures in eight patients. ICU and hospital stays were 6.7 ± 8.1 and 15.7 ± 13.9 days, respectively. Renal replacement therapy (RRT) was required in 16 patients (5.8%) within 48 hours of ICU stay and in 28 patients (10.2%) within 43weeks. Mortality at 28 days was 2.9%. Eighty-six patients (31.4%) were diagnosed with AKI within 48 hours of surgery. Area under the ROC curve of POST uNGAL for AKI diagnosis was 0.72 (0.66 to 0.79) (*P *0.0001) at an optimal cutoff value of 1803 μg/l, with 78.7% specificity, 64% sensitivity and 74.1% accuracy. uNGAL advanced diagnosis of AKI in 44 patients (51.2%), whereas diagnosis was achieved at the same time as AKI criteria in 11 patients; AKI criteria outperformed uNGAL in only 36% of cases. Accordingly, uNGAL was useful to diagnose postoperative AKI in 63.9% of cases. Median delta uNGAL was 12.5 (from -1.9 to 71.1) and 154.5 (from 16.6 to 484.5) μg/l in non-AKI and AKI patients, respectively (*P *0.0001) and its area under the ROC curve for AKI prediction was 0.70 (0.63 to 0.77) (*P *0.0001).

## Conclusion

Compared with AKIN criteria, a urinary NGAL concentration >180 µg/l anticipates AKI diagnosis in more than 50% of cardiac surgery patients in the first 24 to 48 hours after intervention.

